# Nurses' Knowledge, Attitude, and Influencing Factors regarding Physical Restraint Use in the Intensive Care Unit: A Multicenter Cross-Sectional Study

**DOI:** 10.1155/2020/4235683

**Published:** 2020-05-22

**Authors:** Tilahun Kassew, Ambaye Dejen Tilahun, Bikis Liyew

**Affiliations:** ^1^Department of Psychiatry, College of Medicine & Health Sciences, University of Gondar, Gondar, Ethiopia; ^2^School of Nursing, Department of Emergency and Critical Care Nursing, College of Medicine and Health Sciences, University of Gondar, Gondar, Ethiopia

## Abstract

**Background:**

Physical restraint is a common practice in the intensive care units which often result in frequent skin laceration at restraint site, limb edema, restricted circulation, and worsening of agitation that may even end in death. Despite the sensitivity of the problem, however, it is felt that there are nurses' evidence-based practice gaps in Ethiopia. To emphasize the importance of this subject, relevant evidence is required to develop protocols and to raise evidence-based practices of health professionals. So, this study aimed to assess the knowledge, attitude, and influencing factors of nurses regarding physical restraint use in the intensive care units in northwest Ethiopia.

**Methods:**

An institution-based cross-sectional study was maintained from March to September 2019 at Amhara regional state referral hospitals, northwest Ethiopia. A total of 260 nurses in the intensive care units were invited to take part in the study by a convenience sampling technique. The Level of Knowledge, Attitudes, and Practices of Staff regarding Physical Restraints Questionnaire was used to assess the nurses' knowledge and attitude. Linear regression analysis was employed to examine the influencing factors of knowledge and attitude. Adjusted unstandardized beta (*β*) coefficient with a 95% confidence interval was used to report the result of association with a *p* value < 0.05 statistical significance level.

**Result:**

The mean scores of nurses' knowledge and attitude regarding physical restraint use among critically ill patients were 7.81 ± 1.89 and 33.75 ± 6.50, respectively. These mean scores are above the scale midpoint nearer to the higher ranges which imply a moderate level of knowledge and a good attitude regarding physical restraint. Lower academic qualification and short (<2 years) work experience were associated with lower-level of knowledge, and reading about restraint from any source and taken training regarding restraints were factors associated with a higher knowledge. Diploma and bachelor's in academic qualification were significantly associated with a negative attitude regarding restraint. Besides, there was a more positive attitude among nurses with a higher level of knowledge and who received training regarding physical restraint use.

**Conclusion:**

The nurses working in the intensive care unit had a moderate level of knowledge and a good attitude regarding physical restraint use. So, developing and providing educational and in-service training to the nurses regarding physical restraint are necessary to strengthen the quality of care for critically ill patients.

## 1. Introduction

Physical restraint is the action or procedure restricting a person's freedom of movement, physical activity, or normal access to his/her body by the use of any physical or mechanical tools and devices attached to the patient's body [1, 2]. It is commonly used in hospitals especially in the intensive care unit settings when patients' are confused, physically harmful to themselves and others, and when the alternative methods are inadequate or contraindicated [1–3]. Because of the questionable ethical and legal concerns related to patients' right of autonomy and dignity, the physical restraint is a heavy controversial procedure [4]. Around 80% of critically ill patients who are admitted to the intensive care unit (ICU) may require to be physically restrained due to the presence of agitation, confusion, sleeplessness, and disruptive behaviors [5]. These behaviors causes removal of life support medical devices, falling injure, and danger to patient him/herself or others. ICU staffs try to reduce those behaviors by using different measures like settling the attendant with patients, lowering bed height, raising bed rails, and using the sedating medication before physical restraint [6, 7].

However, health professionals in the ICU use physical restraint to prevent interference in treatment procedures and protection of patient's harm. It is highly preferred when chemical restraint is associated with long term sedation and risk of psychosis [1, 5, 8]. Several works of literature reported that the prevalence of physical restraint use among critically ill patients ranged from 62% to 79% worldwide [9–12].

Researches showed that restrained patients in the ICU encounter prolonged hospital stays, and adverse psychological and physical consequences like agitation, aggression, limb edema, and skin laceration at the restraint site, and fall which results in a poor quality of health care [9, 13, 14]. Different studies [15–18] have shown more than half of critically ill patients have restrained and they have faced complications from PR despite insufficient guidelines and regulations on the use of physical restraints. Nurses' knowledge and attitudes regarding PR is an important factor for providing good nursing care for critically ill patients. Nurses with a higher level of knowledge and better attitudes regarding PR contribute to improving their practice on the application of PR and alternative strategies in the treatment of critically ill patients. Again, these result in quality patient care through minimizing physical, psychological, and ethical dilemma problems associated with PR use [5, 19, 20].

Studies reported that the nurses' level of knowledge about the use, purpose, and complications of PR use is low [21, 22]. According to Gastmans and Möhler and Meyer, most nurses' attitudes are negative regarding PR use. Furthermore, the researchers found that nurses had ambivalent feelings, guilt, and frustration when they restrain patients [23–25]. Additionally, most nurses do not believe that using physical restraints in hospitals can result in restlessness, aggression, and injury [26]. Older age, male gender, long years of experience, longer working years at ICU, day shift work, higher academic qualification, reading information about PR in the past year, lower number of patients care per day, and receiving educational and in-service training about PR are the influencing factors for nurses' adequate knowledge and better attitude [10, 22, 25, 27, 28].

In many countries such as the United States [16, 17], several European [15, 23, 29]and Asian countries [21], and a few African countries [28, 30], the application of physical restraints in hospitals has been studied in detail. Even if there are variations to PR use in the ICU settings, those countries recommend evidence-based medical restraints to be used and implemented in hospitals to minimize PR-related adverse effects. Besides, educational training, protocols, and policies have developed and applied for proper documentation and use of restraints in hospitalized patients [18, 20]. In Ethiopia, physical restraint use is a common clinical practice of nursing care in critical care settings to prevent confused patients' interference. The usually used devices for restraining in our setting are ropes, chains, and patient's cloth. Despite these being commonly used, there is no clear regulatory guidance concerning this issue. Additionally, there are no studies regarding nurses' knowledge on and attitude towards physical restraint use for critically ill patients. This assessment is very crucial to explore the need of developing protocols, the nursing education, and training, which help the nurses to have a good practice for PR use. Hence, the aim of this study was to assess the knowledge, attitude, and influencing factors of nurses regarding PR use in the ICU at Amhara regional state referral hospitals, northwest Ethiopia.

## 2. Methods

### 2.1. Study Setting and Population

A multicentered institution-based cross-sectional study was conducted among nurses working in ICU at Amhara Regional State Referral Hospitals (ARSRHs), Northwest Ethiopia, from July to August 2019. There are five referral hospitals (Felege-Hiwot, Dessie, Debre-Markos, Debre-Birhan, and University of Gondar referral hospitals) in which intensive care is provided for critically ill patients. Nurses who were working in neonatal and pediatric ICU, on annual leave during data collection and head nurses, were excluded from the study. Adequacy of sample size was maintained through recruiting all nurses working in adult ICU settings to be participating in the study since the study population was minimal. Hence, all nurses (*N* = 260) working in ICUs in those hospitals during the data collection period were invited to participate in the study for the power of representativeness through the convenience sampling technique. From those, two-hundred and thirty-seven nurses were willing and filled the questionnaire.

### 2.2. Data Collection Procedure and Tool

A structured self-administered questionnaire was used to assess nurses' knowledge, attitude, and influencing factors regarding PR use. The questionnaires comprised three parts: personal and professional characteristics, level of knowledge, and attitude of staff regarding the PR questionnaire. Data were collected by five nurses who were the heads of each hospital's ICU who distributed the questionnaires to the respondents by getting their willingness and collected the filled data. The training was given for data collectors regarding the questionnaire such as how to recruit the participants and how to explain unclear questions and the purpose of the study for participants. Furthermore, the training included information about the ethical principles such as confidentiality, anonymity, secures subjects' informed consent, and recruiting them with their willingness for participation. The questionnaire was prepared by the researchers in the English language which is used for the collection of the information. It was adopted from the previous studies. Two critical care nurses, one nursing educator, and the researchers have examined the questionnaire for face and content validity, clarity, and discrimination of items. The questionnaire was revised and modified appropriately with the local context. A pilot survey among 20 Ethiopian nurses was conducted at Saint's Paulos referral hospital to establish the reliability of the questionnaire. The questionnaire format was filled in their clinical area by the respondent nurses in the presence of the data collectors.

#### 2.2.1. The First Part

The first part took part with personal and professional characteristics of the participant nurses such as gender, age, marital status, academic qualification, work experience in ICU, whether received educational training regarding PR during their graduate class, reading books and articles about PR in the past year, and years of work experience in ICU. We assessed received educational training by the question: “have you received educational training regarding PR during your graduate class?” and the response was yes/no. To examine reading information about PR, we asked respondents: “have you read books and articles about physical restraint in the past year?” and the response was “yes and/no”.

#### 2.2.2. The Second and Third Parts

These parts comprised the knowledge and attitude part of the Level of Knowledge, Attitudes, and Practices of Staff regarding Physical Restraints Questionnaire to measure the nurses' knowledge and attitude regarding physical restraint, respectively. This tool was initially developed by Janelli et al. [31] and then improved by Suen [32] which again was adapted by Kaya et al. in 2008 [33].

The second part of the tool consists of 11 items that include 10 positively worded questions and 1 negatively worded question which measures the knowledge of nurses regarding PR use. The responses to the questions were agree or disagree. The correct answer is scored as 1, and the wrong answer is scored as 0. The total score range of this part is 0–11; nurses with the total score of 11 indicated the highest level of knowledge and those with a total score of 0 indicated a lower level of knowledge. The third part comprised 12 items, which included 4-point Likert scale, that measured the attitudes of nursing staff regarding the use of physical restraints. The responses are “strongly agree” = 4 points, “agree” = 3 points, “do not agree” = 2 points, and “strongly disagree” = 1 with the total score range 12–48. Nurses with a total score closer to 48 represented the best possible attitude and those with a score closer to 12 represented a very poor attitude regarding PR use [31–33]. Cronbach's alpha coefficient in this study was 0.76 and 0.85 for knowledge and attitude, respectively.

### 2.3. Data Processing and Analysis

Coded variables were entered into the Epi-Data version 3.1 and then exported and analyzed using SPSS version-20. Frequency, percentage, mean, standard deviation and range were used to summarize data and evaluate the distribution of variables. After performing assumption tests, simple linear regression was performed to determine the correlation of each independent variable with knowledge and attitude. The independent variables were personal and professional related information such as age, gender, marital status, academic qualifications, whether received training, reading any information about PR, years of work experiences in ICU, working shift time, and observing complications with PR use. The dependent variables are knowledge and attitude scores. The knowledge score was also independent in case of attitude. Variables with *p* value < 0.2 during simple linear regression such as age, sex, academic qualification, reading information about PR in the past year, whether received training about PR in a graduate class, work experience at ICU, and work shift time were selected for further analysis in multiple linear regression, and model fitness tests (adjusted *R*^2^) were also checked. Factors associated with the knowledge and attitude regarding PR use were expressed as adjusted unstandardized *β* coefficient with a 95% confidence interval at a *p* value of < 0.05 statistical significance level.

### 2.4. Ethical Approval and Consent to Participate

Ethical approval was obtained from the Institutional Review Board (IRB) of the University of Gondar. The official letter of cooperation was submitted to all referral hospitals, and then a formal letter of permission was obtained from each hospital. Before data collection, the aim of the study was explained verbally to the participants and after their willingness; written permission was obtained before filling the questionnaire. So, informed written consent was obtained from the participants, and confidentiality was maintained by omitting their identification.

## 3. Results

### 3.1. Personal and Professional Characteristics of the Participant Nurses

A total of 237 participants took part in the study with a response rate of 91.2%. The mean age of the participants was 30.50 ± 9.93 with a range of 23–40 years. Nearly two-thirds of the participants (62.4%) were married, and one-hundred twenty-nine (54.4%) were males. The majority (67.1%) of the participant nurses' had bachelor's degree by their academic qualification. One-hundred and five (44.3%) of the nurses had less than two years of work experience in the ICU. Nearly half of the participants (49.8%) read books/articles about PR in the past year. Eighty-one (34.2%) study participants received educational training during their graduate class, but no one had gotten in-service training regarding restraining. More than half of the nurses (57.4%) were to have complications among restrained patients associated with PR. The most frequent complications noticed by nurses were edema, pain, and bruising around the restrained site. Fatigue, agitation, anger, and aspiration were the other types of observed complications associated with PR use among critically ill patients (Table 1).

### 3.2. Nurses' Knowledge and Attitude regarding Physical Restraint Use in ICU

The mean score of knowledge regarding PR among nurses working in the ICU was 7.81 ± 1.89 (95% CI (7.56, 8.05)) with the range of 4–11. The proportion of the participant nurses' who have scored above the knowledge scale midpoint was 80.9%; this indicated that the majority of nurses' had a moderate level of knowledge regarding PR. In this part, 85.4% of the participants responded correctly for the question, “If physical restraints (safety vest, garment) are to be used, a member of the patient's family is required to sign a consent form” and 78.1% the nurses responded correctly for question “Deaths have been linked to the use of vest restraints” and 71.7% of the nurses responded incorrectly to question “Good alternatives to restraints do not exist” (Table 2).

The mean score of nurses' attitude regarding PR was 33.73 ± 6.50 (95% CI (32.89, 34.56)) with the range of 18–47. The proportion of the participant nurses' who have scored above the attitude scale midpoint was 68.8%; this indicated that the majority of nurses' had a good level of attitude regarding PR. In this part, 80.4% of the nurses agreed (agree + strongly agree) with the sentence “A patient suffers a loss of dignity when placed in restraints.,” and 78.9% of the nurses reported to agree with “I feel that family members have the right to refuse the use of restraints,” whereas nearly half of the nurses, i.e., *N* = 115 (48.5%), disagreed (disagree + strongly disagree) for the question “I feel that placing a patient in restraints can decrease nursing care time” and 43.3% of the participants disagreed to the sentence “I believe that restraints increase the risk of strangulation” (Table 3).

### 3.3. Factors Associated with Nurses' Knowledge and Attitude regarding Physical Restraint Use

The simple linear regression analysis showed that age, sex, academic qualification, reading books about PR in the past year, whether received training about PR in graduate classes, years of work experience in the ICU, and work shift time were factors found to be associated with knowledge regarding PR at *p* value < 0.2. The nurses' knowledge score is the other selected factor in multiple linear regressions for attitude regarding PR in addition to the above factors used for nurses' knowledge. The result of the multiple linear regression indicated that diploma and bachelors in academic qualification, training in a graduate class about PR, and short (<2 years) work experience in ICU were significantly associated with knowledge regarding PR at *p* value < 0.05 (Table 4). Diploma and bachelors in academic qualification, training during graduate class, and knowledge score were the factors significantly associated with nurses' attitude regarding PR at *p* value < 0.05 in multiple linear regression (Table 5).

The knowledge of nurses regarding PR use who had diploma and bachelors by academic qualification is lower by 2.65 [95% CI = −3.46, −1.85] and 1.11 [95% CI = −1.63, −0.59] units, respectively, compared with masters. The Nurses' knowledge regarding PR use is lower by 1.55 units [95% CI = −2.14, −0.96] among nurses with short (<2 years) work experience as compared with those with longer (>5 years) work experience in the ICU. The nurses' knowledge regarding PR use is higher by 2.86 units [95% CI = 1.37, 4.34] among nurses who had read information about PR in the past year as compared with those who had not read any information about PR. The nurses' knowledge regarding PR use is higher by 0.82 units [95% CI = 0.34, 1.29] among nurses who had received training during the graduate class compared with those who had not received educational training.

The nurses' attitude regarding PR use is decreased by 4.42 [95% CI = −7.50, −1.34] and 2.24 [95% CI = −4.15, −0.34] units among diploma and bachelor's as compared with those among masters by academic qualification. The attitude of nurses regarding PR use who had received training regarding PR during graduate class is increased by 2.9 units [95% CI = 1.27, 4.53] as compared with those who had not received educational training. Furthermore, a unit higher in the nurses' knowledge score results in 0.95 units [95% CI = 0.49, 1.41] increase in the nurses' attitude regarding PR use in the ICU.

## 4. Discussion

PR is widely used in intensive care unit settings among critically ill patients' to reduce the risk of a patient's falling, prevent the removal of life support equipment and procedures, and reduce the risk of patients harming himself or among others [1]. Apart from the use of PR in critical care settings, it can result in prolonged hospital stays, complications, and ethical issues as a result of nurses' inadequate knowledge on and negative attitude with improper practices regarding PR use [7, 19, 27, 29] which reduces power from even the most beneficial medical treatments [34]. Improving ICU nurses' knowledge, attitude, and practice on the use of restraining, therefore, is essential to prevent the complications of it and to enhance ICU care services.

This study reflected that the majority of the nurses' had a moderate level of knowledge regarding PR use among critically ill patients in which greater than 80% of the participants had a certain important understanding regarding physical restraint use. This finding is in agreement with other of studies conducted in Sudan and Sakarya, Turkey [28, 35], but higher than those of other studies conducted in India and province of Konya, Turkey [22, 27]. The possible reason for the variation might be attributed to the sociocultural differences and differences between participants. The sociocultural beliefs and values can affect the individuals awareness and perception on their daily activities expressed within the local social environment. In our setting, restraints are culturally practiced and the health care workers are culturally competent that helps them to have adequate understanding regarding restraints which strengthen the nurses to provide appropriate care for restrained patients. In the Indian study, the participants were nurses working in the psychiatry ward. Calming agents are the most preferred and used therapeutic modality to control the patients' abnormal behavior in mental health care settings over PR. Furthermore, psychiatric patients can tolerate the adverse effects of chemical restraints compared with critically ill patients [6, 36]. As a result, nursing staffs in the ICU settings may have more information regarding restraining due to their frequent usage. On the other hand, the level of nurses' knowledge regarding PR of this study is lower than that of the studies held in Egypt [25], Jordan [10], and Malaysia [21]. In those studies, most of the participants had received in-service training, participants were ICU specialized nurses, and the hospitals had a guideline about the use of restraint that may influence their knowledge to have higher; on the contrary, in our study, most of the participants were comprehensive nurses working in the ICU with no one who received in-service training and weak guideline on restraint in the hospitals that result in a lower knowledge in contrast to others.

This study reflected that majority of the nurses' had a more positive attitude regarding PR use among the ICUs in which greater than 68% of the participants had a certain important positive belief and attitudes regarding physical restraint use. The level of the nurses' attitude in this study is consistent with that of other studies [25, 35] but higher than some other studies [28, 37] in which majority of the nurses had a negative attitude. The possible reason for the variation might be attributed to the sample size differences and differences characteristics of participants'. In this study, the participants were 237 qualified nurses working in the ICU, whereas the participants were small in number (below one hundred) and working in other settings in which the application of PR is minimal compared with ICU in the counterpart studies. So, this finding is supposed to have a more positive attitude regarding PR use. On the other hand, the level of nurses' attitude regarding PR in this study is lower than in other studies [21, 27]. In the previous studies, most of the participants were ICU specialized nurses who also had received training and the study setting had a good guideline about the use of restraint that may result to have a better attitude; on the contrary, in our setting, no one has received training and a weak policy of hospitals on the restraint that resulted in a certain negative belief and attitudes.

Regarding influencing factors: short (<2 years) work experience in ICU was significantly associated with inadequate knowledge regarding PR use but not correlated with attitude score. The possible reason might be when the nurses had fewer years of work experience, they challenged to comprehend and apply the previously learned information regarding PR use as required which improved through experience than those with higher years of experience [38, 39]. This finding was consistent with that of a previous study [28]. This study found a significant association of academic qualification with nurses' knowledge and attitude regarding PR use. Nurses' with a diploma and bachelor's degree had lower knowledge and poor attitude regarding PR use as compared with nurses qualified by master's science. This finding is consistent with that of other studies [15, 21]. The possible reason for this association might be nurses with a diploma and bachelor's degree are more comprehensive and received less training on restraining during graduate class as a procedure in fundamental and other courses of nursing [40].

This finding indicated that receiving training about PR during graduate class was significantly associated with a higher level of knowledge and a better attitude of nurses regarding PR use. This finding is consistent with that of other studies [21, 25] which showed adequate knowledge and positive attitudes regarding PR use were correlated with taken up training concerning the issue. Training is the process of transforming information and skills through learning for trainees related to restraining that influences the nurses to have better awareness and attitude and performs proper clinical practice regarding restraining of critically ill patients. This shows that effective educational programs about PR use via appropriate guidelines are needed to maximize the nurses' understanding related to patient's rights and autonomy and ethical and legal aspects of the restraining and restraint alternatives [25, 41, 42]. Furthermore, our finding revealed that nurses who had read about restraint from any source in the past year had a higher level of knowledge regarding PR compared with nurses' who had not read about it. This is consistent with a previous similar study [21]. This possibly because reading about PR from any source is one way of learning about restraint that can change knowledge by expanding and updating their understanding and awareness about PR use.

Additionally, our study founded that the nurses' attitude was influenced by their level of knowledge regarding PR use. Nurses with a higher level of knowledge had an association with a better attitude regarding PR use. This result is in agreement with the previous works of the literature [15, 21, 25]. The possible reason might be based on the theory of planned behavior [43, 44]; knowledge is important for the basis of nurses' behavior to affect their subjective feeling regarding restraint use and perform a proper nursing activity during constraints. So, pieces of training concerning PR use for nursing staff are necessary to improve their knowledge and attitude related to PR among critically ill patients [30].

The participants were nurses who were motivated by their willingness to participate in the study, which limits the external generalizability of the result. Besides, the research could not show the cause-effect relationship between predictors, and knowledge and attitude owing to its cross-sectional nature.

## 5. Conclusion

The nurses' working in the ICU had a moderate level of knowledge and a good attitude regarding PR use among critically ill patients. However, a higher level of knowledge and a better attitude of nurses regarding the subject is needed. It revealed that recruiting experienced and more qualified nurses in the ICU and providing educational training about the use of PR are crucial to strengthen the quality of care for critically ill patients. Indeed, improving nurses' knowledge results in a more positive feeling and beliefs; they transform their knowledge and belief to proper nursing practice in the application of PR. It also recommended to conduct an observational study to notice whether training and years of work experience can improve the nurses' knowledge and attitude regarding PR use.

## Figures and Tables

**Table 1:** Personal and professional characteristics of nurses working in ICU at Amhara regional state referral hospitals, 2019 (*n* = 237).

Variables	Categories	Frequency	Percentage (%)
Sex	Male	129	54.4
	Female	108	45.6
Participants' religion	Orthodox	181	76.4
	Muslim	43	18.1
	Protestant	13	5.5
Marital status	Unmarried	71	30.0
	Married	148	62.4
	Divorced	18	7.6
Academic qualification	Diploma	25	10.5
	Bachelor's	159	67.1
	Master's	53	22.4
How long you have worked as a nurse (years of work as a nurse)?	<5 years	94	39.7
	5–10 years	118	49.8
	>10 years	25	10.5
How long you have worked in the ICU (years of work in the ICU)?	<2 years	105	44.3
	2–5 years	87	36.7
	>5 years	45	19.0
Have you used PR in the past month?	Yes	96	40.5
	No	141	59.5
Have you read about PR from any source (like books, articles…) in the past year?	Yes	118	49.8
	No	119	50.2
Have you received educational training regarding PR in your graduate class?	Yes	81	34.2
	No	156	65.8
Use of alternative methods before PR	Yes	125	52.7
	No	112	47.3
Number of patient care per day	2 patients	59	24.9
	3-4 patients	122	51.5
	≥5 patients	56	23.6
Observing complications from PR	Yes	136	57.4
	No	101	42.6
Working shift time	Day shift	66	27.8
	Night shift	35	14.8
	Day and/night shift	136	57.4
Age	Range 23–40	Mean = 30.5	SD = 9.93
		Median 30	IQR = 28–33

**Table 2:** Participant nurses' knowledge response regarding physical restraint use at Amhara regional state referral hospitals, 2019 (*n* = 237).

Items	Responses *n* (%)	
	Correct	Incorrect
(1) Physical restraints are safety garments designed to prevent injury	158 (66.7)	79 (33.3)
(2) Restraints should be used when one cannot watch the patient closely	153 (64.6)	84(35.4)
(3) Patients are allowed to refuse to be placed in a restraint	169 (71.3)	68 (28.7)
(4) If physical restraints (safety vest and garment) are to be used, a member of the patient's family is required to sign a consent form	202 (85.2)	35 (14.8)
(5) Restraint should be released every 2 hours if the patient is awake	175 (73.8)	62 (26.2)
(6) Restraints should be put on tightly so that there is no space between the restraint and the patient's skin	162 (68.4)	75 (31.6)
(7) When a patient is restrained, the skin can break down or restlessness can increase	153 (64.6)	84 (35.4)
(8) When a patient is restrained in bed, the restraint should not be attached to the side rail	153 (64.6)	84 (35.4)
(9) A patient should never be restrained while lying flat in bed because of the danger of choking	172 (72.6)	65 (27.4)
(10) Good alternatives to restraints do not exist	67 (28.3)	170 (71.7)
(11) Deaths have been linked to the use of vest restraints	185 (78.1)	52 (21.9)

Knowledge total mean score with standard deviation	7.81 ± 1.89; 95% CI: (7.56–8.05)	Range = 4–11, 80.9% above the scale midpoint score

**Table 3:** Participant nurses' attitude response regarding physical restraint use at Amhara regional state referral hospitals, 2019 (*n* = 237).

	Responses *n* (%)			
	Strongly agree	Agree	Disagree	Strongly disagre
(1) I feel that family members have the right to refuse the use of restraints	82 (34.6)	105 (44.3)	29 (12.2)	21 (8.9)
(2) If I were the patient, I feel I have the right to refuse or resist when restraints are placed on me	74 (31.2)	85 (35.9)	60 (25.3)	18 (7.6)
(3) I feel discomfort/guilt when placing a patient on restraint	50 (21.1)	107 (45.1)	73 (30.8)	7 (3.0)
(4) I feel that the main reason restraints are used is that the hospital is short-staffed	47 (19.8)	81 (34.2)	90 (38.0)	19 (8.0)
(5) I feel embarrassed when the family enters the room of a restrained patient and they have not been informed	68 (28.7)	78 (32.9)	74 (31.2)	17 (7.2)
(6) It makes me feel bad if the patients get more upset after restraints are applied	67 (28.3)	83 (35.0)	79 (33.3)	8 (3.4)
(7) It makes me feel bad when patients become more disoriented after the restraints have been applied	70 (29.5)	99 (41.8)	55 (23.2)	13 (5.5)
(8) A patient suffers a loss of dignity when placed in restraints	61 (25.9)	129 (54.5)	39 (16.2)	8 (3.4)
(9) The hospital is responsible for adhering to the laws on the use of restraints to ensure the safety of a patient	60 (25.3)	114 (48.2)	48 (20.2)	15 (6.3)
(10) I Feel that placing a patient in restraints can decrease nursing care time	51 (21.5)	71 (30.0)	97 (40.9)	18 (7.6)
(11) I Believe that restraints increase the risk of strangulation	45 (19.0)	89 (37.60	73 (30.8)	30 (12.7)
(12) In general, I feel knowledgeable about caring for a restrained patient	39 (16.5)	122 (51.5)	54 (22.8)	22 (9.3)

Attitude total mean score with standard deviation	33.73 ± 6.50; 95% CI: (32.89–34.56)		Range = 18–47 (68.8% above the scale midpoint cutoff point)	

SA = strongly agree; A = agree; D = disagree; SD = strongly disagree.

**Table 4:** Factors associated with nurses' knowledge regarding physical restraint in multiple linear regression analysis (*n* = 237).

Variables	Categories	Knowledge (sum score)	
		Crude unstandardized *β* coefficient (95% CI)	Adjusted unstandardized *β* coefficient (95% CI)
Sex	Male	0	0
	Female	−1.43 (−1.88, −0.98)	0.28 (−1.04, 1.59)
Academic qualification	Master's	0	0
	Bachelor's	−0.75 (−1.26, −0.24)	−1.11 (−1.63, −0.59)^∗^
	Diploma	−2.20 (−2.94, −1.46)	−2.65 (−3.46, −1.85)^∗∗^
Reading about PR in the past year	No	0	0
	Yes	5.95 (4.47, 7.43)	2.86 (1.37, 4.34)^∗^
Received training in a graduate class about PR	No	0	0
	Yes	1.70 (1.24, 2.17)	0.82 (0.34, 1.29)^∗∗^
Work experience at ICU	>5 years	0	0
	2–5 years	0.34 (−0.16, 0.85)	−0.49 (−1.04, 0.07)
	<2 years	−1.52 (−1.96, −1.07)	−1.55 (−2.14, −0.96)^∗∗∗^
Work shift time	Regular day	0	0
	Shift day or night	−0.41 (−0.90, 0.08)	−0.13 (−0.56, 0.30)
	Shift day and night	−0.54 (−1.23, 0.14)	−0.23 (−0.81, 0.35)
Age of nurses	In years	0.16 (0.09, 0.23)	0.01 (−0.06, 0.07)

0 = reference, ^*∗*^*p* < 0.05, ^*∗∗*^*p* < 0.01, ^*∗∗∗*^*p* < 0.001. Adjusted *R*^2^ = 46.9%, *F* test *p* value < 0.001.

**Table 5:** Factors associated with nurses' attitude regarding physical restraint use in multiple linear regression analysis (*n* = 237).

Variables	Categories	Attitude (sum score)	
		Crude unstandardized *β* coefficient (95% CI)	Adjusted unstandardized *β* coefficient (95% CI)
Sex	Male	0	0
	Female	−2.52 (−4.09, −0.95)	−0.76 (−2.21, 1.30)
Academic qualification	Master's	0	0
	Bachelor's	−2.57 (−4.31, −0.83)	−2.24 (−4.15, −0.34)^∗^
	Diploma	−7.07 (−9.63, −4.52)	−4.42 (−7.50, −1.34)^∗∗^
Reading about PR in the past year	No	0	0
	Yes	1.20 (0.74, 1.66)	0.61 (−1.14, 2.35)
Received training in a graduate class about PR	No	0	0
	Yes	5.71 (4.11, 7.30)	2.90 (1.27, 4.53)^∗∗^
Work experience in the ICU	>5 years	0	0
	2–5 years	0.47 (−1.27, 2.20)	0.44 (−1.52, 2.39)
	<2 years	−3.58 (−5.19,−1.96)	−0.50 (−2.69, 1.68)
Work shift time	Regular day	0	0
	Shift day or night	−2.15 (−3.81,−0.49)	−1.24 (−2.73, 0.26)
	Shift day and night	−1.15 (−3.50, 1.19)	−0.91 (−2.99, 1.17)
Age of nurses	In years	0.52 (0.29, 0.76)	0.14 (−0.1, 0.35)
Knowledge	Total-score	1.91 (1.55, 2.28)	0.95 (0.49, 1.41)^∗∗∗^

0 = reference, ^*∗*^*p* < 0.05, ^*∗∗*^*p* < 0.01, ^*∗∗∗*^*p* < 0.001. Adjusted *R*^2^ = 43.9%, *F* test *p* value < 0.001.

## Data Availability

The data sets used in the study are available from the corresponding author on reasonable request (tilahunkassew123@gmail.com).

## Abbreviations

ARSRHs: Amhara Regional State Referral Hospitals
ICU: Intensive Care Unit
PR: Physical restraint
SPSS: Statistical Packages of Social Sciences.
